# Supplementary material for: Solution structure of mouse HBS1L/SKI7-specific UBA domain in complex with ubiquitin: Implications for stalled ribosome recognition
Source: PLoS One. 2026 Jun 3;21(6):e0348877. doi: 10.1371/journal.pone.0348877 (PMC13232801; doi:10.1371/journal.pone.0348877)
Supplement: S1 Table — (PDF) [file pone.0348877.s001.pdf]

S1 Table. Completeness of resonance assignments in the UBAh domain and its complex with ubiquitin at pH 6.0 and 298 K.

Assignment completeness (%)	Free UBAh	UBAh in complex with Ubiquitin	Ubiquitin in complex with UBAh
$1H^N$, $13C^\alpha$ and $15N^H$ backbone[a]	99.5%	99.0%	97.7%
13C'	95.7%	100.0%	98.7%
$1H^\alpha$	98.6%	98.6%	100.0%
All side-chain proton and corresponding carbon and nitrogen resonances[b]	99.8%	96.7%	99.6%
BMRB accession number	36786	36787	36787

[a] Excluding Pro residues.

[b] Excluding non-protonated ^{13}C chemical shifts of aromatic rings, OH protons of Ser/Thr, ε-amino groups of Lys residues, and guanidinium groups of Arg residues.
