# Supplementary material for: Solution structure of mouse HBS1L/SKI7-specific UBA domain in complex with ubiquitin: Implications for stalled ribosome recognition
Source: PLoS One. 2026 Jun 3;21(6):e0348877. doi: 10.1371/journal.pone.0348877 (PMC13232801; doi:10.1371/journal.pone.0348877)
Supplement: S2 Table — (PDF) [file pone.0348877.s002.pdf]

S2 Table. Summary of conformational constraints and structural statistics for 20 energy-refined conformers of UBAh in its free form.

**NMR distance and dihedral angle constraints**	
Distance restraints	
Total NOE	906
Sequential ($\|i-j\| = 1$)	490
Medium-range ($1 < \|i-j\| < 5$)	242
Long-range ($\|i-j\| \geq 5$)	174
φ/ψ dihedral angle restraints (TALOS)	81
χ dihedral angle constraints	0
**Structure statistics**	
AMBER energies (kcal/mol)	
Total	–3,429.45 ± 14.50
Distance restraints	2.90 ± 0.32
Dihedral angle restraints	0.52 ± 0.44
Ramachandran plot statistics (%)	
Residues in most favored regions	91.4
Residues in additionally allowed regions	8.4
Residues in generously allowed regions	0.2
Residues in disallowed regions	0.0
Average RMSD from mean coordinates (Å):	
Backbone (Res. 70–118)	0.55 ± 0.18
Heavy atoms (Res. 70–118)	1.24 ± 0.18
