# Supplementary material for: Solution structure of mouse HBS1L/SKI7-specific UBA domain in complex with ubiquitin: Implications for stalled ribosome recognition
Source: PLoS One. 2026 Jun 3;21(6):e0348877. doi: 10.1371/journal.pone.0348877 (PMC13232801; doi:10.1371/journal.pone.0348877)
Supplement: S1 Fig — (PDF) [file pone.0348877.s003.pdf]

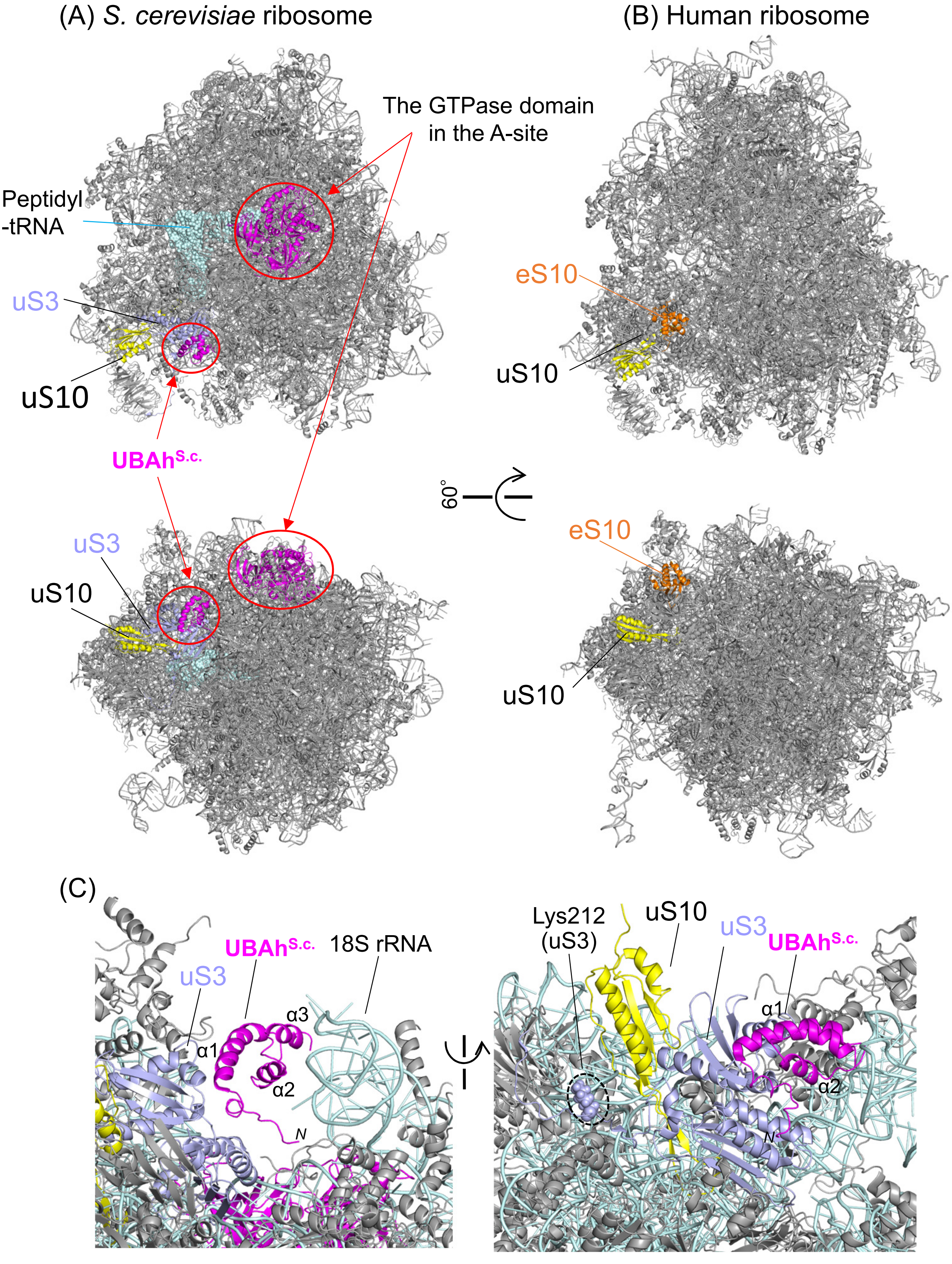

S1 Fig. (legend on next page)

S1 Fig. Structure of eukaryotic 80S ribosomes.

(A) *S. cerevisiae* non-stop ribosome in complex with the Hbs1–Dom34–GTP complex [PDB ID 5M1J]. The Hbs1 in the complex is shown in magenta. Notably, the GTPase domain of HBS1 in the complex binds to the A-site of the 60S subunit, whereas $UBAh^{S.c.}$ binds to the 40S subunit. The region connecting the two structural domains is disordered and therefore invisible. $Phe\text{-}tRNA^{Phe}$ in the P-site is shown in light blue. uS10 and uS3, which undergo ubiquitination, are shown in yellow and purple, respectively. The figure in the upper panel is shown rotated 60° in the direction indicated by the arrow from the lower panel.

(B) Human 80S ribosome [8QOI]. uS10 and eS10, which undergo ubiquitination, are shown in yellow and orange, respectively.

(C) Close-up view of the $UBAh^{S.c.}$ interaction with the 40S subunit. $UBAh^{S.c.}$ fits snugly into a cavity of the 40S subunit. Ubiquitination also occurs on uS3 (at Lys212, displayed in a sphere model for clarity) but not in humans. The right panel shows the left panel rotated in the indicated orientation.
