# Supplementary material for: Solution structure of mouse HBS1L/SKI7-specific UBA domain in complex with ubiquitin: Implications for stalled ribosome recognition
Source: PLoS One. 2026 Jun 3;21(6):e0348877. doi: 10.1371/journal.pone.0348877 (PMC13232801; doi:10.1371/journal.pone.0348877)
Supplement: S2 Fig — (PDF) [file pone.0348877.s004.pdf]

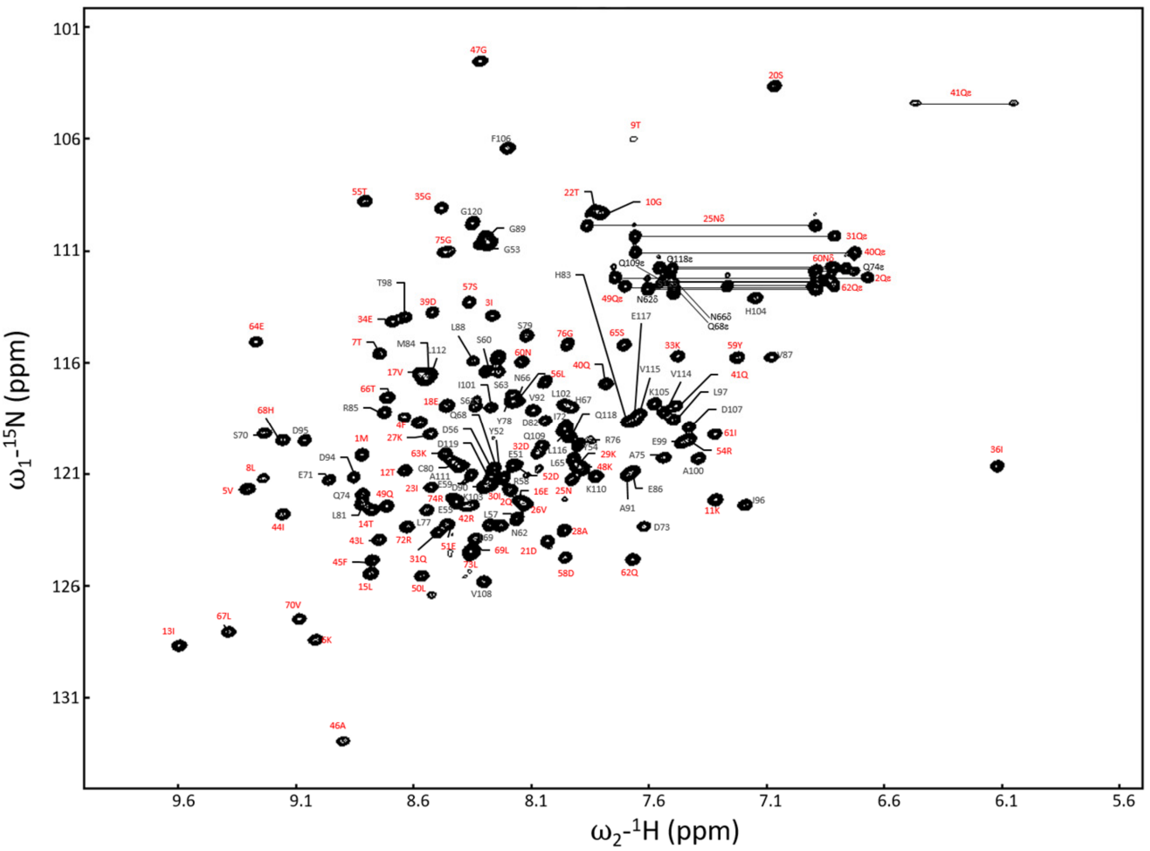

S2 Fig. Assigned 2D ^{1}H-^{15}N HSQC spectrum of the UBAh domain in the presence of ubiquitin (1:1 molar ratio).

Data were acquired on a Bruker 800 MHz spectrometer using the echo-antiecho method. The assignments of UBAh and ubiquitin are labeled in black and red, respectively.
