# Supplementary material for: Solution structure of mouse HBS1L/SKI7-specific UBA domain in complex with ubiquitin: Implications for stalled ribosome recognition
Source: PLoS One. 2026 Jun 3;21(6):e0348877. doi: 10.1371/journal.pone.0348877 (PMC13232801; doi:10.1371/journal.pone.0348877)
Supplement: S3 Fig — (PDF) [file pone.0348877.s005.pdf]

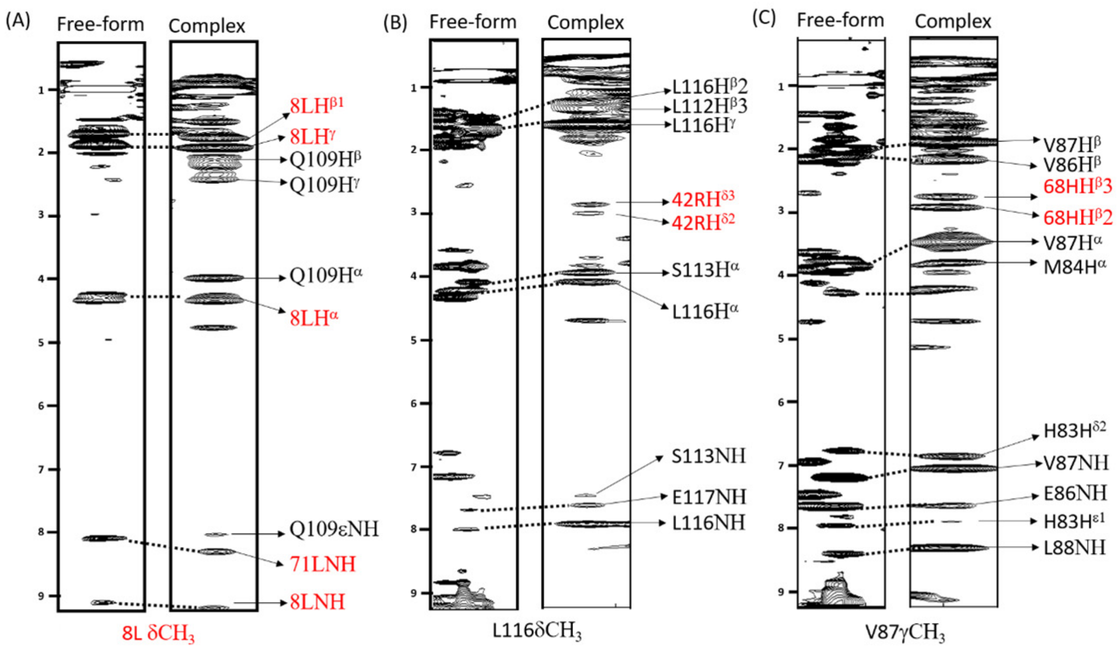

S3 Fig. Representative intermolecular NOE strip plots illustrating contacts between UBAh and ubiquitin (2D strips from a 3D ^{13}C-edited NOESY spectrum).

(A) NOE strip plots derived from the δ-methyl resonance of Leu8 in ubiquitin in the absence and presence of UBAh (left and right panels, respectively). Peaks originating from UBAh and ubiquitin are indicated by black and red labels, respectively.

(B, C) NOE strip plots derived from the δ-methyl resonances of Leu116 and Val87 in UBAh in the absence and presence of ubiquitin (left and right panels, respectively).
