# Supplementary material for: Solution structure of mouse HBS1L/SKI7-specific UBA domain in complex with ubiquitin: Implications for stalled ribosome recognition
Source: PLoS One. 2026 Jun 3;21(6):e0348877. doi: 10.1371/journal.pone.0348877 (PMC13232801; doi:10.1371/journal.pone.0348877)
Supplement: S4 Fig — (PDF) [file pone.0348877.s006.pdf]

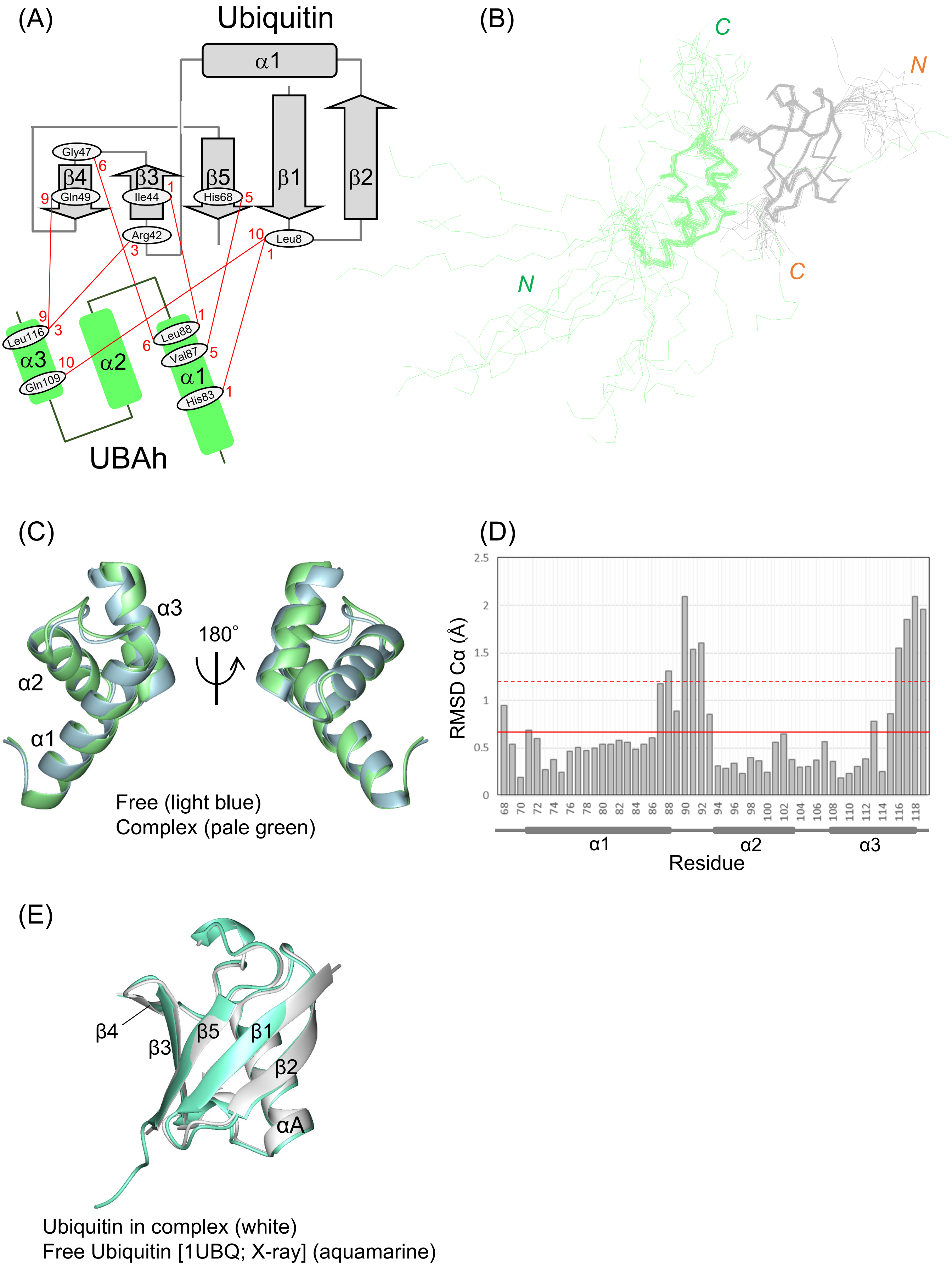


**S4 Fig.** (legend on next page)

**S4 Fig. Solution structure of the UBAh–ubiquitin complex and comparison of UBAh in its free and bound forms.**

(A) Schematic diagram of intermolecular NOEs between UBAh and ubiquitin. The NOEs are shown as red lines with the corresponding numbers. A total of 35 NOEs were observed between the two molecules.

(B) A trace of the backbone atoms for the 20 superimposed lowest-energy conformers of the complex (the N-terminal tag, UBAh, and the C-terminal tag; the N-terminal tag and ubiquitin). The pale green and light gray lines represent the Cα traces of UBAh and ubiquitin, respectively.

(C) Ribbon representation of the superimposed lowest-energy UBAh structures in free and complex forms (fitting region: residues 68–119). The RMSD for the backbone atoms (N, Cα, C′) is 0.825 Å.

(D) Per-residue Cα RMSD between the lowest-energy structures of free and complex UBAh, plotted against residue number. The red line indicates the mean RMSD value, and the red dotted line indicates the mean + 1 standard deviation. Residues with values above the red dotted line are considered to exhibit significant structural differences between free and complex UBAh.

(E) Ribbon representation of superimposed ubiquitin structures (residues 1–72) from this study and from the X-ray structure of free ubiquitin [1UBQ]. The RMSD for the backbone atoms is 0.606 Å.
