# Supplementary material for: Solution structure of mouse HBS1L/SKI7-specific UBA domain in complex with ubiquitin: Implications for stalled ribosome recognition
Source: PLoS One. 2026 Jun 3;21(6):e0348877. doi: 10.1371/journal.pone.0348877 (PMC13232801; doi:10.1371/journal.pone.0348877)
Supplement: S5 Fig — (PDF) [file pone.0348877.s007.pdf]

(A)

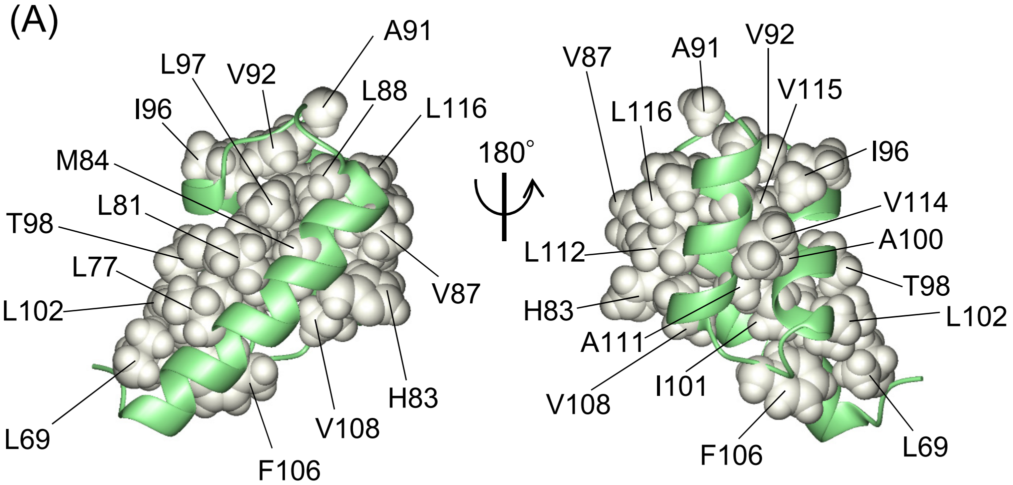

(B)

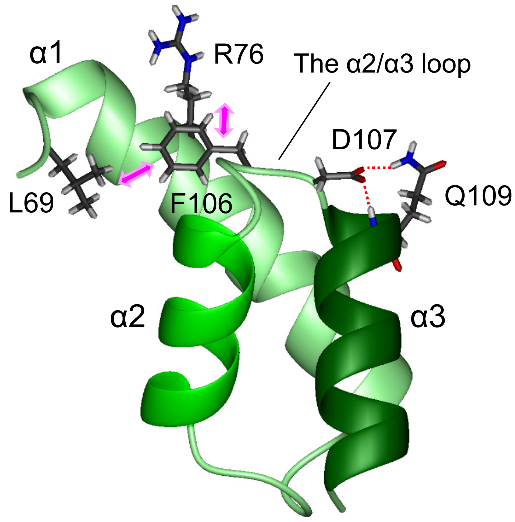

S5 Fig. Structural features of UBAh.

(A) Large hydrophobic core of UBAh. The CPK representation of the core is superimposed on the ribbon model. Residues that form this core include: Leu69 upstream of α1; Leu77 Leu81, His83, Met84, Val87, and Leu88 in α1; Ala91 and Val92 in the α1/α2 loop; Ile96, Leu97, Thr98 (via its methyl group), Ala100, Ile101, and Leu102 in α2; Phe106 in the α2/α3 loop; and Val108, Ala111, Leu112, Val114, Val115, and Leu116 in α3. Only residues that can be indicated with arrows are labeled in the figure.

(B) Roles of residues in the α2/α3 loop (^{104}HKFD^{107}). The aromatic ring of Phe106 in the loop engages in hydrophobic interactions with the aliphatic portion of the Arg76 side chain in α1 and with the Leu69 side chain in the upstream region of α1, as indicated by the wide pink arrows. The side chain of Asp107 in the loop forms a hydrogen bond with the backbone amide and/or the amide nitrogen of Gln109, thereby serving as an N-terminal helix cap of α3, as indicated by red dotted lines.
