# Supplementary material for: Solution structure of mouse HBS1L/SKI7-specific UBA domain in complex with ubiquitin: Implications for stalled ribosome recognition
Source: PLoS One. 2026 Jun 3;21(6):e0348877. doi: 10.1371/journal.pone.0348877 (PMC13232801; doi:10.1371/journal.pone.0348877)
Supplement: S6 Fig — (PDF) [file pone.0348877.s008.pdf]

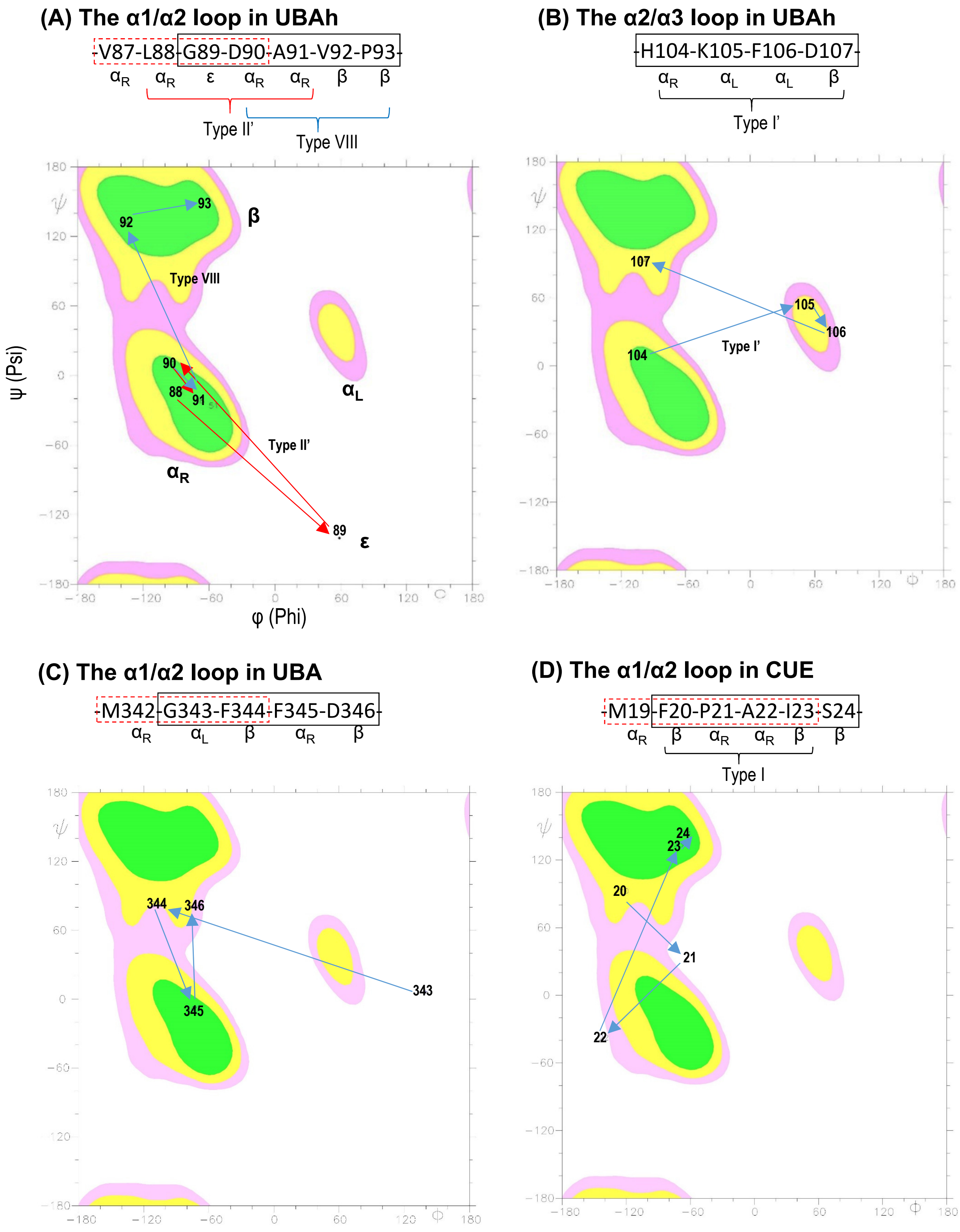

S6 Fig. Ramachandran plots of residues forming the α1/α2 (A) and α2/α3 loop (B) in UBAh, the α1/α2 loop (C) in UBA [1WR1], and the α1/α2 loop (D) in CUE [1OTR]. Black boxes indicate the loop sequences of each domain and red dotted boxes indicate the hallmark sequences of each domain. The types of β-turns are indicated in (A), (B), and (D), whereas the loop in UBA (C) does not contain a canonical β-turn. A comparison of the α1/α2 loop structures is provided in Fig 5F.
