# Supplementary material for: Solution structure of mouse HBS1L/SKI7-specific UBA domain in complex with ubiquitin: Implications for stalled ribosome recognition
Source: PLoS One. 2026 Jun 3;21(6):e0348877. doi: 10.1371/journal.pone.0348877 (PMC13232801; doi:10.1371/journal.pone.0348877)
Supplement: S7 Fig — (PDF) [file pone.0348877.s009.pdf]

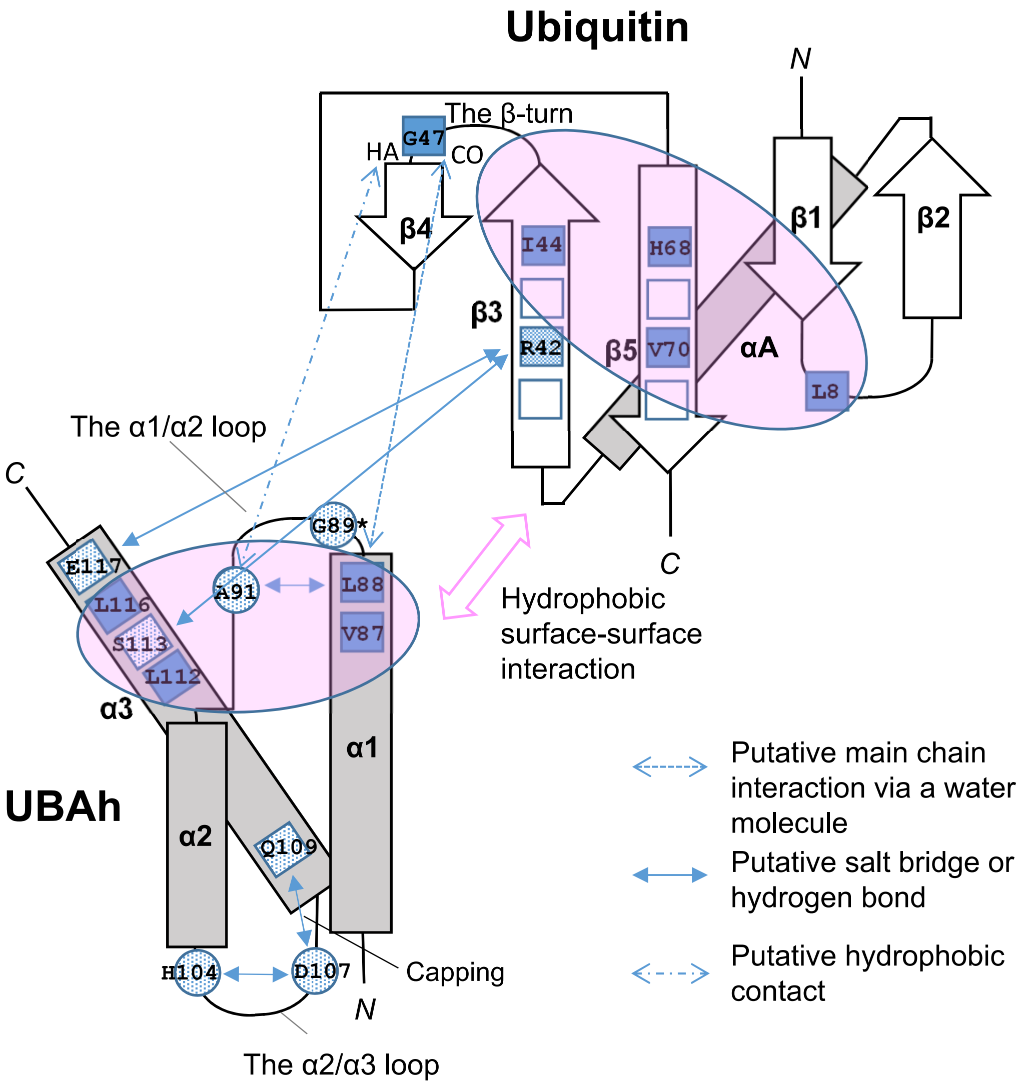

S7 Fig. Schematic diagram of the interactions between UBAh and ubiquitin.
Residues involved in the interactions and the characteristic residues forming the UBAh structure are indicated. Different types of interactions are represented by distinct arrow styles as shown in the figure. For Gly89*, the absence of a side chain is important to avoid steric clashes with the β-turn of ubiquitin.
