# Supplementary material for: Solution structure of mouse HBS1L/SKI7-specific UBA domain in complex with ubiquitin: Implications for stalled ribosome recognition
Source: PLoS One. 2026 Jun 3;21(6):e0348877. doi: 10.1371/journal.pone.0348877 (PMC13232801; doi:10.1371/journal.pone.0348877)
Supplement: S9 Fig — (PDF) [file pone.0348877.s011.pdf]

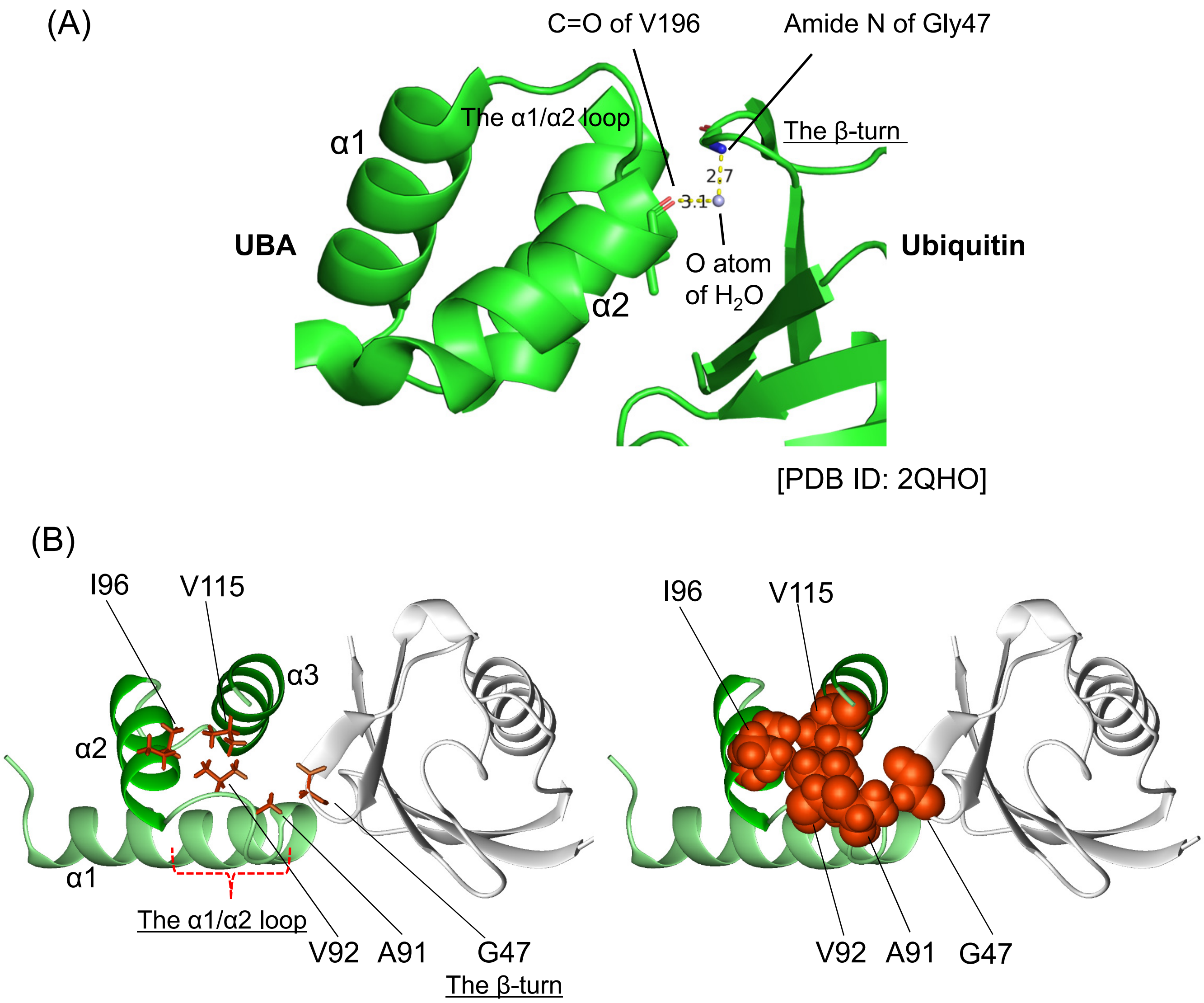

S9 Fig. Characteristic interaction sites between UBAh and ubiquitin.

(A) Example of a water-mediated interaction between the C=O of a hydrophobic residue at the N-terminus of α2 and the N–H of Gly47 in ubiquitin, as seen in the X-ray complex structure of the UBA of ubiquitin ligase EDD with ubiquitin [2QHO]. This figure was generated by PyMOL.

(B) Roles of the characteristic structure of the α1/α2 loop. Left, involved residues shown in the ribbon representation of the UBAh–ubiquitin complex; right, the same residues shown in a CPK model. The side chain of Ala91 forms a van der Waals contact with the Hα proton of Gly47 in ubiquitin, whereas that of Val92 points inward to engage in hydrophobic interactions with those of Ile96 in α2 and Val115 in α3.
