# Supplementary material for: Solution structure of mouse HBS1L/SKI7-specific UBA domain in complex with ubiquitin: Implications for stalled ribosome recognition
Source: PLoS One. 2026 Jun 3;21(6):e0348877. doi: 10.1371/journal.pone.0348877 (PMC13232801; doi:10.1371/journal.pone.0348877)
Supplement: S10 Fig — (PDF) [file pone.0348877.s012.pdf]

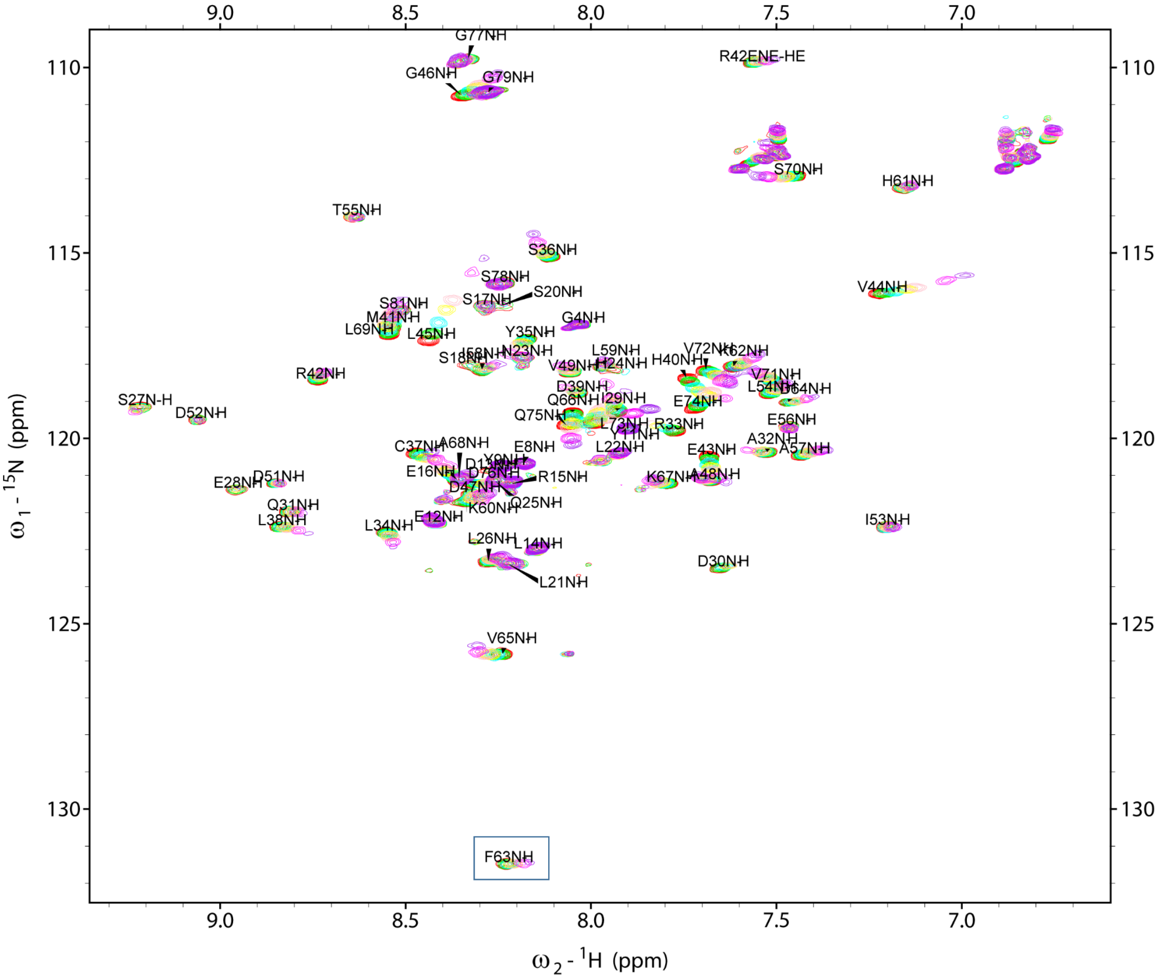


```
Red: without ubiquitin
Green:   1.0 : 0.1
Blue:    1.0 : 0.2
Yellow:  1.0 : 0.5
Pink:    1.0 : 1.0
Magenta: 1.0 : 2.0
Purple:  1.0 : 3.0
```

S10 Fig. [^{1}H, ^{15}N]-HSQC spectra of labeled UBAh at different ubiquitin concentrations. UBAh was used at an initial concentration of 100 µM, and ubiquitin was added in six increments, ranging from 0.1- to 3.0-fold molar equivalents, as shown. The addition of 43 to the displayed residue numbers yields the corresponding positions in the full-length protein. The resonance derived from the NH group of Phe63 (boxed) appears as folded peaks.
