# Supplementary material for: Solution structure of mouse HBS1L/SKI7-specific UBA domain in complex with ubiquitin: Implications for stalled ribosome recognition
Source: PLoS One. 2026 Jun 3;21(6):e0348877. doi: 10.1371/journal.pone.0348877 (PMC13232801; doi:10.1371/journal.pone.0348877)
Supplement: S11 Fig — (PDF) [file pone.0348877.s013.pdf]

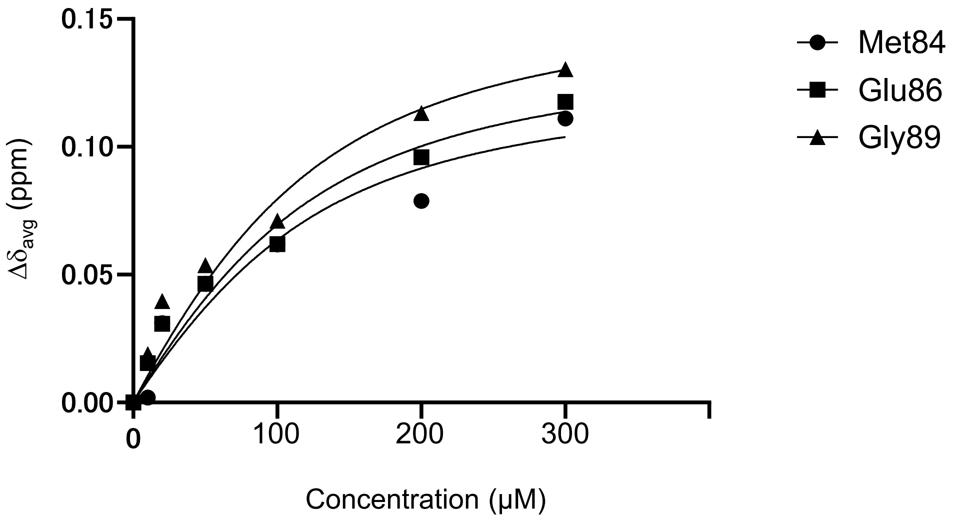

S11 Fig. Chemical shift perturbation titration curves for the interaction between ^{15}N-labeled UBAh and unlabeled ubiquitin.

The weighted chemical shift changes for three residues (Met84 and Glu86 in α1 and Gly89 in the α1/α2 loop) of UBAh are plotted against the ubiquitin concentration (μM). The curves were obtained by global nonlinear regression with GraphPad Prism 6 using a single-site binding model (the formula is provided in the Materials and Methods). This analysis yielded a Kd of 51.6 μM.
