# Supplementary material for: Solution structure of mouse HBS1L/SKI7-specific UBA domain in complex with ubiquitin: Implications for stalled ribosome recognition
Source: PLoS One. 2026 Jun 3;21(6):e0348877. doi: 10.1371/journal.pone.0348877 (PMC13232801; doi:10.1371/journal.pone.0348877)
Supplement: S13 Fig — (PDF) [file pone.0348877.s015.pdf]

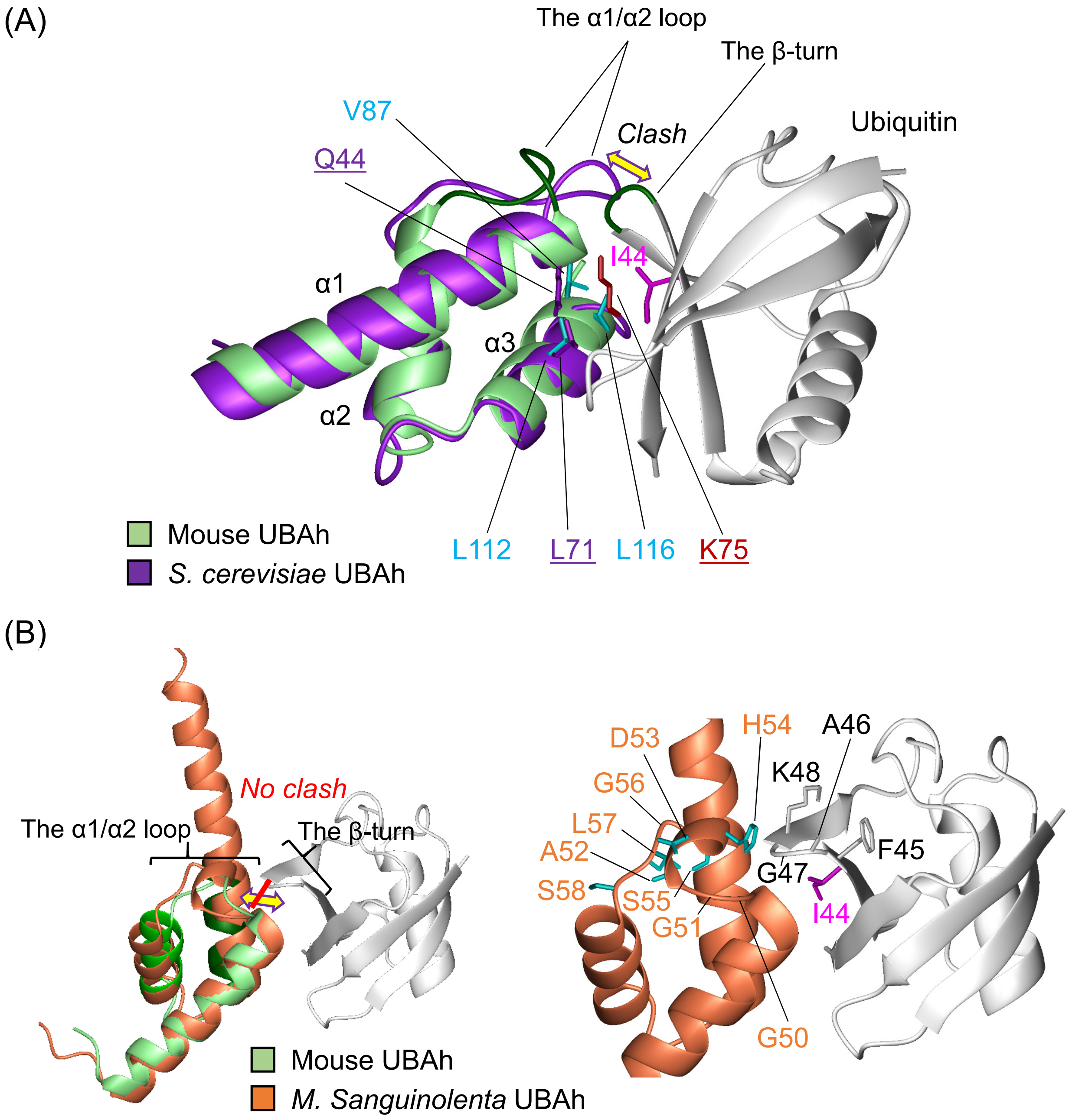

S13 Fig. Structural comparison of mouse UBAh (from the UBAh–ubiquitin complex) between other UBAh structures.

(A) Superposition of mouse UBAh and *S. cerevisiae* UBAh$^{s.c.}$. The ribbon representation of *S. cerevisiae* UBAh is shown in purple, derived from the Hbs1–Dom34 complex with a stalled ribosome [PDB ID: 5M1J]. In the mouse complex, UBAh and ubiquitin are shown in pale green and light gray, respectively. Residues involved in the interaction are highlighted in mouse UBAh (V87, L112, and L116), with the corresponding residues indicated in *S. cerevisiae* UBAh (Q44, L71, and K75). Ile44 is shown only in ubiquitin.

(B) Superposition of mouse UBAh and *M. sanguinolenta* UBAh. Left: The ribbon representation of *M. sanguinolenta* UBAh is shown in orange, predicted by AlphaFold. Right: Close-up view of the α1/α2 loops. The residues forming the loops are indicated.
