# Supplementary material for: Solution structure of mouse HBS1L/SKI7-specific UBA domain in complex with ubiquitin: Implications for stalled ribosome recognition
Source: PLoS One. 2026 Jun 3;21(6):e0348877. doi: 10.1371/journal.pone.0348877 (PMC13232801; doi:10.1371/journal.pone.0348877)
Supplement: S14 Fig — (PDF) [file pone.0348877.s016.pdf]

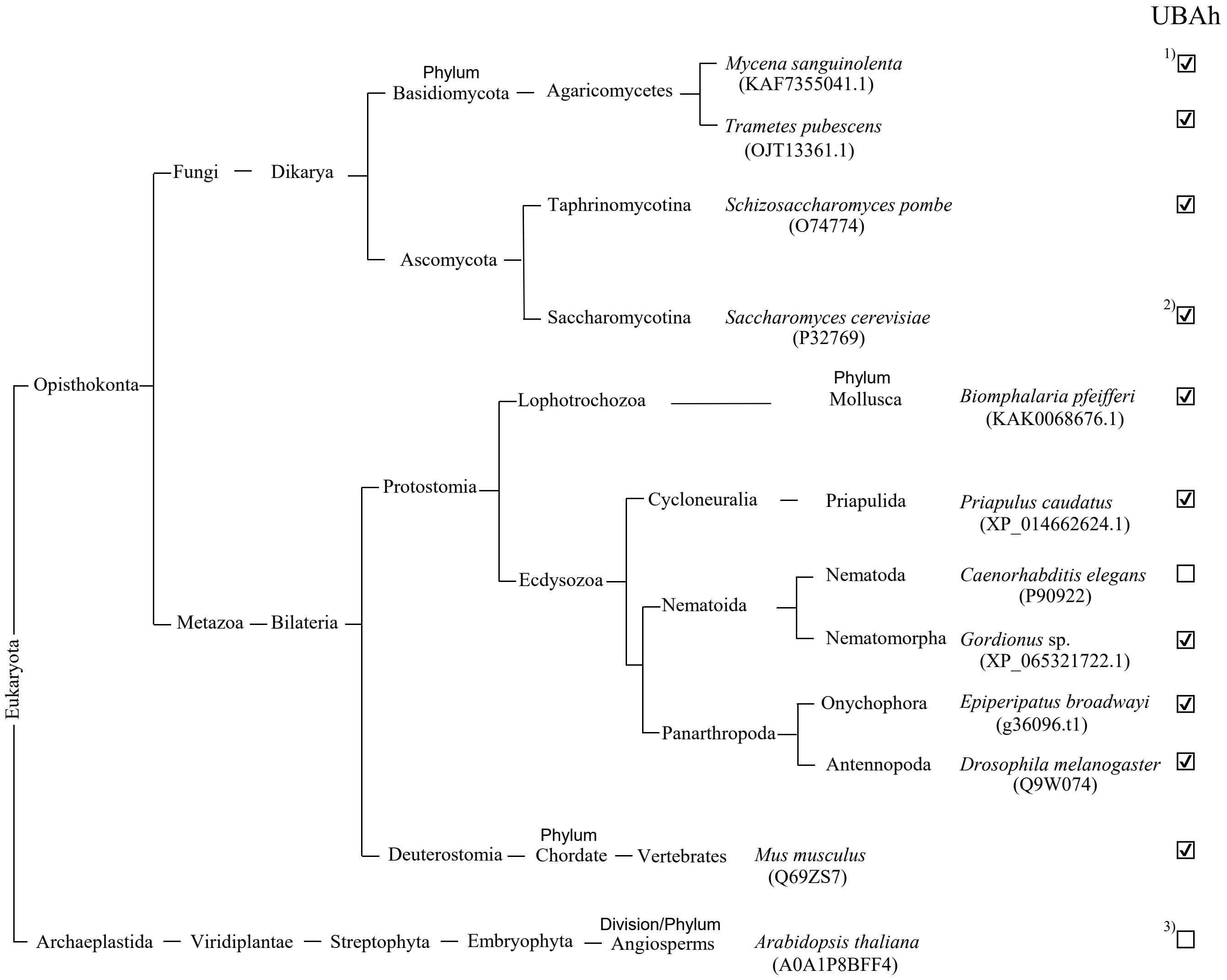

1) This UBAh domain has an α1/α2 loop that is 3–4 residues longer than those in most species but still contains the UBAh-specific motif. This domain appears capable of binding ubiquitin.
2) The UBAh from *S. cerevisiae* is non-canonical and thus appears unable to bind ubiquitin.
3) In most plants, HBS1L orthologs possess a zinc-finger domain in place of UBAh, which is of the ubiquitin-binding type.

S14 Fig. Simplified phylogenetic tree of major eukaryotes indicating the presence of UBAh in HBS1L.

A tree was constructed as described by Burki et al. (Burki et al., 2020). The checkmark in each box indicates the presence of UBAh in the HBS1L orthologs of the indicated species. Accession codes are shown below the species names. Although SKI7 contains UBAh in many species, particularly in all vertebrates, SKI7 orthologs in some species, such as *S. cerevisiae*, lack this domain (see Kalisiak et al. for details) (Kalisiak et al., 2017).

Burki F, Roger AJ, Brown MW, Simpson AGB. The New Tree of Eukaryotes. Trends Ecol Evol. 2020;35(1):43-55.
