# Supplementary material for: Solution structure of mouse HBS1L/SKI7-specific UBA domain in complex with ubiquitin: Implications for stalled ribosome recognition
Source: PLoS One. 2026 Jun 3;21(6):e0348877. doi: 10.1371/journal.pone.0348877 (PMC13232801; doi:10.1371/journal.pone.0348877)
Supplement: S8 Fig — (PDF) [file pone.0348877.s010.pdf]

UBAh

Domain	Species	Accession codes	PDB ID	Start	Sequence	End
HBS1L/Hbs1	*M. m.*	Q69ZS7		70	SEIDQARLYSCLDHMREVLGDAVPDDILTEAILKHKFDVQKALSVVLEQD	119
	H. s.	Q9Y450		74	SGFDQARLYSCLDHMREVLGDAVPDEILIEAVLKNKFDVQKALSGVLEQD	123
	G. g.	E1BW59		78	TEVDRARLSSCLDQMREVLAESVPDQAMVQAVLDSKFDVQKALDLVLSQG	127
	C. p.	A0A8C3HXA3		77	TGVDQARLYSCLDQMREVLGETAPEQVMVEAVLNSKFDVQKALDLVLAQD	126
	X. l.	Q6GNS6		76	SAVDQARLYSCLDHMREVLGESVMEQVMIDAVLKCQFDVAKALDLVFKQD	125
	D. r.	F1Q5Z6		79	DSLQQGRLYSCLDQMRTVLGDSIPDSTLTQAALKYDCDPHRALDFILSEN	128
	D. m.	Q9W074		93	DEIEQAKLSSCVDEVRSVVGDAVSERRIVETSMKFDYDMQKILDEILNEE	142
	S. p.	O74774		28	EEQEEEFRSAVATVRETLLGVPISEKEIADTVWYYYFDVEKSVNYLLQKA	77

UBA

Domain	Species	Accession codes	PDB ID	Start	Sequence	End
Dsk2	*S. c.*	P48510	1WR1/4UN2	327	PPEERYEHQLRQLNDMGFF--DFDRNVAALRRSGGSVQGALDSLLNGD	372
Ubiquilin-1	*H. s.*	Q9UMX0	2JY6	542	NPEVRFQQQLEQLSAMGFL--NREANLQALIATGGDINAAIERLLGSQ	587
BMSC-UbP	*H. s.*	Q96S82	2DEN	333	SLQSQWQPQLQQLRDMGIQ--DDELSLRALQATGGDIQAALELIFAGG	378
NBR1	*H. s.*	Q14596	2MJ5	912	ISEDQTAALMAHLFEMGFC--DRQLNLRLLKKHNYNILQVVTELLQLN	957
Ube2K	*H. s.*	P61086	6IF1	157	VSSPEYTKKIENLCAMGF---DRNAVIVALSSKSWDVETATELLSN	200
Ddi1	*H. s.*	P40087	2MRO	386	TGRTFPEQTIKQLMDLGF---PRDAVVKALKQTNGNAEFAASLLFQ	428

CUE

Domain	Species	Accession codes	PDB ID	Start	Sequence	End
Cue2-1 CUE	*S. c.*	P36075	1OTR	4	DNNDDHESKLSILMDMFPAI-SKSKLQVHLLENNNDLDLTIGLLLKEN	50
CUE in ASCC2	*H. s.*	Q9H1I8	2DI0	463	MCGVELDSLISQVKDLLPDL-GEGFILACLEYYHYDPEQVINNILEER	509
CUEDC1	*H. s.*	Q9NWM3	2DHY	42	VRRLEFNQAMDDFKTMFPNM-DYDIIECVLRANSGAVDATIDQLLQMN	88
Tollip	*H. s.*	Q9H0E2	1WGL	225	AQPRCSEEDLKAIQDMFPNM-DQEVIRSVLEAQRGNKDAAINSLLQMG	271
RUH-075	*H. s.*	Q9Y679	2EKF	292	SPDVQLATLAQRVKEVLPHV-PLGVIQRDLAKT-GCVDLTITNLLEGA	337
gp78 CUE	*H. s.*	Q9UKV5	2LVN	452	ASNSQLNAMAHQIQEMFPQV-PYHLVLQDLQLT-RSVEITTDNILEGR	497

S8 Fig. Amino acid sequence alignments of UBAh, UBA, and CUE domains.

Alignments were generated using ClustalW (Larkin et al., 2007) and manually adjusted based on the structure of each domain. The alignment of UBAh is identical to that shown in Fig 2B; sequences from *M. sanguinolenta* and *S. cerevisiae* were omitted. Accession codes for proteins containing each domain and PDB IDs of the domains are provided. Underlined PDB IDs indicate complexes with ubiquitin. Hydrophobic residues conserved within each domain are highlighted in light beige, key residues interacting with ubiquitin are shown in bold red (Fig 5), and the hallmark motifs of each domain are boxed.
