# Supplementary material for: Solution structure of mouse HBS1L/SKI7-specific UBA domain in complex with ubiquitin: Implications for stalled ribosome recognition
Source: PLoS One. 2026 Jun 3;21(6):e0348877. doi: 10.1371/journal.pone.0348877 (PMC13232801; doi:10.1371/journal.pone.0348877)
Supplement: S12 Fig — (PDF) [file pone.0348877.s014.pdf]

```
Numbering         1 2 3 4 5 6 7 8 9 10 11 12 13 14 15 16 17 18 19 20   21 22 23 24 25 26 27 28 29 30 31 32 33 34 35 36 37 38 39 40 41 42 43 44 45 46 47 48   49 50 51 52 53 54 55 56 57 58 59 60 61 62   63 64 65 66 67
M. musculus       M A R H R N V R G Y N Y D E D F E D D D - L Y G Q S V E D D Y C I S P S T A A Q F I Y S R R D N P - - - - - - - - - - - - - - - - - - - - - - E E E Y G Y E D L R E S S N - - S L L N H 67
H. sapiens        M A R H R N V R G Y N Y D E D F E D D D - L Y G Q S V E D D Y C I S P S T A A Q F I Y S R R D K P S - - - - - - - - - - - - - - - - - - V E P V E E Y D Y E D L K E S S N - - S V S N H 71
G. gallus         M S R H R N V R G Y N Y D E D F E D D D - V Y G Q S V E D D Y C I S P S T A A Q F I Y S R R D K P A T - - - - - - - - - - - - - - - F A E P L E E E Y G Y E D T E D T A G - Y F T S N H 75
C. picta bellii   M A R H R N V R G Y N Y D E D F E D D D - L Y G Q S V E D D C C I S P S T A A Q F I Y S R R D K P S A - - - - - - - - - - - - - - - F A - E R L E E Y G Y E D A E E P A K - N I T S N H 74
X. laevis         M A R H R N V R G Y N Y D D D F D D D D - L Y G Q S V E D D Y C I S P A T A A Q F I Y K R D R Q T S F T - - - - - - - - - - - - - - E P L E E E E E G E Y E E L D K - - - - L K A T D S 73
D. rerio          M S R H R N V R G Y N Y D E D F E D D D - M Y G Q S V E D D Y C I S P A T A A Q F I Y S R Q D S R Q A - - - - - - - - - - - - - - - R H V E T V E E A E Y E E E E E E M P T S P T M T S 76
D. melanogaster   M S R H R I V R T M D Y N D E Y D G Y D D I Y G H S V E D E H C I S P T D A Q Q W L Y D R A R G Q Q S I S A F I S K N K D I Q E E E A D E D E D E D A A F A K A R R D S E - - S F Q M P 90
Gordionus sp.     M S R H K N F R G K K F E A E E D D Y D D D Y D D Y E Y S V S P D A S N D F L N P Q S I K I P L S S F E - - - - - - - - - - - - - - - - - - - - - - - - - - - - - - - - - - K H P V I A 58
S. pombe          M S R H R D V K N L D L - D D Y E L D E E P G E E E - - - - - - - - - - - - - - - - - - - - - - - - - - - - - - - - - - - - - - - - - - - - - - - - - - - - - - - - - - - - - - - - - - 25
M. sanguinolenta M V A R H K L V K N M S L D D Q L E D D A L S D G G E D - - - - - - - - - - - - - - - - - - - - - - - - - - - - - - - - - - - - - - - - - - - - - - - - - - - - - - - - - - - - - - - - - 28
S. cerevisiae     M A Y S D Y S D G A D D M P D F H D E G E F D D - - - - - - - - - - - - - - - - - - - - - - - - - - - - - - - - - - - - - - - - - - - - - - - - - - - - - - - - - - - - - - - - - - - - 24
C. elegans        M S R H R A I R N L N L E D E M D D D Y D D D Y D D Y E D E E N P E K Q Y T Y D R N S L S T N Y Y T Y L T D S G R N S P A L P A P H P A P T T S L S S T I V R N M T A P L L H Q N Q K P 92
```

S12 Fig. Amino acid alignment of the N-terminal region upstream of the UBAh domain of HBS1L/SKI7 from various from various eukaryotes.

The alignment was generated using ClustalW (Larkin et al., 2007) and was manually adjusted only in minor parts. Residue numbering above the alignment follows that of mouse HBS1L (682 residues in total); residues 1–67 are shown here, whereas residues 68–120 are presented in Fig. 2B. Asp (D) and Glu (E) residues are shown in red, whereas Ser (S), Thr (T), and Tyr (Y) residues are shown in green. Because *C. elegans* HBS1L lacks the UBAh domain, its sequence starts from the first residue and is not aligned to the others. Accession codes are provided in the Materials and Methods.
