# Supplementary material for: Solution structure of mouse HBS1L/SKI7-specific UBA domain in complex with ubiquitin: Implications for stalled ribosome recognition
Source: PLoS One. 2026 Jun 3;21(6):e0348877. doi: 10.1371/journal.pone.0348877 (PMC13232801; doi:10.1371/journal.pone.0348877)
Supplement: Attachment — Submitted filename: Response to Reviewers_ver3.docx [file pone.0348877.s019.docx]

**Journal Requirements:**

*When submitting your revision, we need you to address these additional requirements.*

***1.*** *Please ensure that your manuscript meets PLOS ONE's style requirements, including those for file naming. The PLOS ONE style templates can be found at*

*https://journals.plos.org/plosone/s/file?id=wjVg/PLOSOne_formatting_sample_main_body.pdf and*

[*https://journals.plos.org/plosone/s/file?id=ba62/PLOSOne_formatting_sample_title_authors_affiliations.pdf*](https://journals.plos.org/plosone/s/file?id=ba62/PLOSOne_formatting_sample_title_authors_affiliations.pdf)

We confirm that our manuscript meets the *PLOS One* style requirements, including those regarding file naming.

***2.*** *We note that this submission includes NMR spectroscopy data. We would recommend that you include the following information in your methods section or as Supporting Information files:*

*(1) The make/source of the NMR instrument used in your study, as well as the magnetic field strength. For each individual experiment, please also list: the nucleus being measured; the sample concentration; the solvent in which the sample is dissolved and if solvent signal suppression was used; the reference standard and the temperature.*

The information has already been described in the previous version. In this revised version, we have explicitly specified the instruments used to improve clarity.

*(2) A list of the chemical shifts for all compounds characterised by NMR spectroscopy, specifying, where relevant: the chemical shift (δ), the multiplicity and the coupling constants (in Hz), for the appropriate nuclei used for assignment.*

The chemical shift assignments of the amino acid residues (¹H, ¹⁵N, and ¹³C) have been deposited in the Biological Magnetic Resonance Data Bank (BMRB) (accession numbers 36786 and 36787), where the complete chemical shift list will be available after publication. The completeness of the assignments is summarized in S1 Table, and the BMRB accession numbers are provided in the Materials and Methods section.

*(3)The full integrated NMR spectrum, clearly labelled with the compound name and chemical structure.*

*We also strongly encourage authors to provide primary NMR data files, in particular for new compounds which have not been characterised in the existing literature. Authors should provide the acquisition data, FID files and processing parameters for each experiment, clearly labelled with the compound name and identifier, as well as a structure file for each provided dataset. See our list of recommended repositories here:* [*https://journals.plos.org/plosone/s/recommended-repositories*](https://journals.plos.org/plosone/s/recommended-repositories)

This paper presents a typical NMR structure determination study using ¹⁵N- and/or ¹³C-labeled proteins composed of the 20 standard amino acids. Therefore, novel chemical compounds were not investigated in this study. NMR chemical shift assignments for the protein were deposited in the Biological Magnetic Resonance Data Bank (BMRB) (accession numbers 36786 and 36787), and the NOE lists used for structure calculations were also deposited **in the Protein Data Bank (PDB)** (accession numbers 9WPR and 9WPS).

Moreover, we have added the full 2D ¹H–¹⁵N HSQC spectrum of the UBAh domain in the presence of ubiquitin (1:1 molar ratio), with peaks labeled with the corresponding amino acid residues (S2 Fig), as well as representative intermolecular NOE strip plots illustrating contacts between UBAh and ubiquitin (2D strips from a 3D ¹³C-edited NOESY spectrum) (S3 Fig).

***3.*** *Thank you for stating the following financial disclosure:*

*“This work was supported by JSPS KAKENHI (grant number 22K06105, to K.K.) and a Daigaku Tokubetsu Kenkyuhi grant from Musashino University (to Y.M. and K.K.).”*

*Please state what role the funders took in the study. If the funders had no role, please state: "The funders had no role in study design, data collection and analysis, decision to publish, or preparation of the manuscript."*

*If this statement is not correct you must amend it as needed.*

*Please include this amended Role of Funder statement in your cover letter; we will change the online submission form on your behalf*

As requested, we have included the following statement in the cover letter: ‘The funders had no role in the study design, data collection and analysis, decision to publish, or preparation of the manuscript.’

***4.*** *When completing the data availability statement of the submission form, you indicated that you will make your data available on acceptance. We strongly recommend all authors decide on a data sharing plan before acceptance, as the process can be lengthy and hold up publication timelines. Please note that, though access restrictions are acceptable now, your entire data will need to be made freely accessible if your manuscript is accepted for publication. This policy applies to all data except where public deposition would breach compliance with the protocol approved by the research ethics board. If you are unable to adhere to our open data policy, please kindly revise your statement to explain your reasoning and we will seek the editor's input on an exemption. Please be assured that, once you have provided your new statement, the assessment of your exemption will not hold up the peer review process.*

We agree with the journal’s open data policy and will make all relevant data freely available upon publication.

***5.*** *If the reviewer comments include a recommendation to cite specific previously published works, please review and evaluate these publications to determine whether they are relevant and should be cited. There is no requirement to cite these works unless the editor has indicated otherwise.*

The reviewers’ comments did not include any recommendation to cite specific previously published works.

-------------------------------------------------------------------------------------------------------------

**5. Review Comments to the Author**

*Please use the space provided to explain your answers to the questions above. You may also include additional comments for the author, including concerns about dual publication, research ethics, or publication ethics. (Please upload your review as an attachment if it exceeds 20,000 characters)*

First, we thank the reviewers for their insightful and constructive comments, which have greatly improved the quality of our manuscript.

We apologize for the oversight regarding the β-turn type of the α1/α2 loop (previously described as type IVb; now corrected to type VIII). We have corrected this in the revised manuscript.

For readability, we have divided the subsection “Structural basis of the UBAh–ubiquitin interaction” in the Results section into two subsections: “Structural basis of the UBAh–ubiquitin interaction: comparison with UBA and CUE domains” and “Distinct α1/α2 loop conformations in UBAh, UBA, and CUE domains: functional implications”, without altering the content.

***Reviewer #1:*** *In this manuscript, the authors structurally characterized a murine HBS1L/SK17-specific UBA domain complexed with ubiquitin using solution NMR spectroscopy. This manuscript presents new structural data of the ubiquitin-binding domain (termed UBAh) of HBS1L/SK17, thereby providing valuable insights into the molecular mechanism mediated by ubiquitin-binding proteins. This reviewer would recommend this manuscript for publication in PLoS ONE. However, there are several concerns that should be adequately addressed during the manuscript revision.*

*Comments:*

*1. Comparing the results presented in the manuscript, the introduction seems overly detailed, which makes the main points difficult to grasp. Specifically, the section from page 7, line 8 (from the bottom), to page 9, line 2 (from the top), is appropriate. However, the preceding section, spanning approximately four pages, is quite lengthy. It would be helpful if the authors could refine this section to be more concise and focused.*

The length appears to be due to the inclusion of long legends for Figs 1 and 2, in accordance with the *PLOS One* guidelines. As mentioned, the Introduction is still lengthy. Therefore, we have shortened the preceding section from 970 to 666 words (the total length of the Introduction is now within three pages, excluding legends). In addition, Fig 1 has been revised to improve clarity.

*2. The authors determined the solution structures of UBAh in its free form and in complex with ubiquitin and subsequently conducted a structural comparison. The authors concluded that ubiquitin binding induces negligible structural changes in UBAh. However, as shown in Figure S4C, the alpha3 helix involved in ubiquitin interaction appeared to be significantly tilted, in contrast to the alpha1 and alpha2 helices. Although the overall RMSD value is reported as 0.825A, presenting a graph showing the RMSD for each individual amino acid residue would allow for a more objective assessment.*

Thank you for your valuable suggestion. Our analysis indicates that α3 is significantly tilted upon ubiquitin binding. We have added a new graph showing the per-residue Cα RMSD (S4E Fig) and included the following sentences in the manuscript:

Page 13, line 22: A comparison of the free and ubiquitin-bound forms of UBAh showed that significant structural changes upon ubiquitin binding occur in the α1/α2 loop and at the C-terminal ends of α1 and α3, as described below (S4D Fig).

Page 17, line 12: This network likely contributes to the positional shift of α3 relative to α1 and α2 upon ubiquitin binding (S4C and S4D Fig)

Page 19, line 17: Furthermore, as a similar pattern was observed in the Cα RMSD values between the free and ubiquitin-bound UBAh structures (S4C Fig), UBAh likely undergoes subtle conformational changes in α1 and α3 relative to α2 upon ubiquitin binding.

*3. The authors used a modified ubiquitin with extra 7AA N-terminal tag for the NMR spectroscopy experiments. To this reviewer's knowledge, most of the (mono)ubiquitin structural biology studies have used non-tagged ubiquitin. The N-termini is close to K63 and is also a modification site for linear Ub chains. Therefore, the authors should show a functional integrity of the modified Ub as compared with the authentic Ub. For example, this could be done by performing a chemical shift perturbation experiment using a commercially available ubiquitin, or by introducing reference example(s) utilizing a modified mono-ubiquitin with an N-terminal tag.*

Nelson et al. performed an NMR-based structural comparison of untagged ubiquitin (residues 1–76, wild type) and its variants containing either a Strep tag plus (GS)₂ or a (GS)₂ tag [9]. In that study, backbone residual dipolar coupling (RDC) analyses of ubiquitin fused to either a Strep tag or (GS)₂ demonstrated that the tagged ubiquitin adopts the same fold in solution as wild-type ubiquitin. Additionally, comparison of chemical shifts between wild-type ubiquitin and (GS)₂-tagged ubiquitin revealed perturbations primarily at Q2 (Δδ_H/N ≈ 0.75 ppm), S57 (≈ 0.13 ppm), and E18 (≈ 0.25 ppm), and to a lesser extent at K63 (≈ 0.05 ppm). The weighted chemical shift difference was calculated as Δδ_H/N = { (Δδ_HN)² + (0.154 × Δδ_N)² }^1/2.

Together, the affected residues clustered within part of the β-sheet and on one side of the α1 helix, centered around the N-terminus (Met1) of ubiquitin, suggesting minor structural changes in this region. Since this region is located on the diametrically opposite face of the protein from the Ile44 hydrophobic patch, the presence of a 7-residue tag is unlikely to substantially alter the interaction mode of UBAh with ubiquitin.

Thus, we have added the following sentences:

Page 13, line 26: Finally, with regard to the ubiquitin structure bearing the N-terminal seven-residue tag, the NMR results indicate…….

Page 13, line 30: N-terminally tagged ubiquitin has been reported to function properly in structure–function studies [52] and in the determination of complex structures [53]. However, as reported by Nelson et al. [52], slight chemical shift differences between tagged and untagged ubiquitin were confined to a limited region near the N-terminus (Met1), specifically within part of the β-sheet and on one side of αA, suggesting minor structural changes in this region. Because this region lies on the face opposite the Ile44 hydrophobic patch, the seven-residue tag is unlikely to substantially affect the UBAh–ubiquitin interaction mode.

**52.**	Nelson SL, Li Y, Chen Y, Deshmukh L. Avidity-Based Method for the Efficient Generation of Monoubiquitinated Recombinant Proteins. J Am Chem Soc. 2023; 145(14): 7748-7752. https://doi.org/10.1021/jacs.3c01943. PMID: 37010382.

**53.**	Lv Z, Rickman KA, Yuan L, Williams K, Selvam SP, Woosley AN, et al. *S. pombe* Uba1-Ubc15 Structure Reveals a Novel Regulatory Mechanism of Ubiquitin E2 Activity. Mol Cell. 2017; 65(4): 699-714.e696. https://doi.org/10.1016/j.molcel.2017.01.008. PMID: 28162934.

***Reviewer #2:*** *In this manuscript, Nameki and He et al. presents a high-quality solution NMR structures of the apo and ubiquitin-bound mouse HBS1L/SKI7-specific UBA domain. The NMR data are rigorous and well-determined, and the interface analysis is convincing. The conclusions are largely appropriate, provided that functional implications are framed with appropriate caution.*

*Overall, this work provides a valuable structural basis for understanding how HBS1L/SKI7 may engage ubiquitin during stalled ribosome surveillance. However, the conceptual framing of UBAh as a "new member" of the THB–Ub group requires more evidence or discussion, and the functional interpretations should be more cautiously phrased before publication.*

*Major comments:*

*1.*

*The authors define UBAh as a distinct member of the THB–Ub group based on a specific analysis of the turn types in the α1/α2 and α2/α3 loops. But, this classification needs more cautious discussion:*

*The authors noted that the turn configurations define this specific type. However, several residues in these loops—such as Phe93, His104, and Lys105 (Fig. 2B)—are not highly conserved across species. These variations could alter the turn geometry in non-mammalian homologs. The authors should provide a more detailed comparative structural analysis (eg. via https://consurf.tau.ac.il/) to support the claim that these turn types are a defining feature of "UBAh" across its entire evolutionary range. Otherwise, the claim should be restricted to mammalian UBAh.*

As suggested, we revised the manuscript to provide a more detailed description of the loops and their conservation among species (page 14, lines 19, 27; page 15, line 11).

Unfortunately, the recommended website was inaccessible. Instead, we used the DALI server for structural comparison and confirmed that the structure of the α1 helix–α1/α2 loop–α2 helix element, including the sequence, is unique to UBAh domains. Additionally, we have clarified that it remains unclear whether the turn geometry of the α2/α3 loop is conserved across eukaryotes.

Page 15, line 1: Furthermore, DALI analysis [55], based on the α1–α1/α2 loop–α2 region, indicated that the α1/α2 loop is uniquely characteristic of UBAh domains in both length and architecture among loops connecting two α-helices, including those of the UBA and CUE domains. Accordingly, this loop serves as a hallmark feature distinguishing the UBAh domain from the UBA and CUE domains.

Page 15, line 15: However, because the residues at positions i and i+1 are not conserved, it remains unclear whether the type I′ β-turn conformation is widely conserved across eukaryotes. Nevertheless, given that the putative turn length was consistently four residues, the overall turn conformation could be maintained.

*On the other hand, if such specific turn-type criteria are used to define a new domain class, could existing UBA or CUE domains also be subdivided into more specific categories (e.g., "UBAc" for CUE)? The authors should discuss whether UBAh is uniquely specialized or if they are proposing a more granular classification system for the entire THB–Ub group. Last, In Fig. S9, the authors compare UBA and CUE domains between yeast and humans. It would be beneficial to also include alignments showing the inter-species conservation of UBA and Cue.*

As pointed out, we agree that existing UBA and CUE domains could potentially be further subdivided into more specific categories based on β-turn types. However, because these domain names are well established and widely used in the field, redefining and systematically reorganizing their classifications requires a more comprehensive analysis. We believe that this is beyond the scope of this study.

UBA and CUE domains are found in various proteins and are often present within the same protein, whereas UBAh is, to our knowledge, exclusively present in HBS1 orthologs. Taken together, these observations suggest that UBAh is uniquely specialized in the recognition of ubiquitin-bound ribosomes.

We have added the following sentences:

Page 20, line 2: These two domains are found in various proteins and are often present within the same protein, whereas UBAh is, to our knowledge, exclusively present in HBS1 orthologs. Thus, within the THB–Ub group, UBAh appears to be uniquely specialized for HBS1-mediated recognition of ubiquitin bound to ribosomes. This study also suggests that the classification of THB–Ub group members may be further refined based on the sequence and structural features of loops containing distinct types of β-turns. It would be of interest to examine why HBS1L orthologs employ a single UBAh domain rather than UBA or CUE domains—that is, why different proteins selectively utilize distinct members of the THB–Ub group.

Sequence alignments illustrating the interspecies conservation of the UBA and CUE domains have been reported in several previous studies. For example, (i) UBA domains described by Ohno et al. (2005) (Structure 13, p. 521, Fig 1), Mueller et al. (2002) (J. Mol. Biol. 319, p. 1243, Fig 5) and Hofmann and Bucher (1996) (FEBS Letters. 21, p. 172, Fig 1), (ii) UBA and CUE domains described by Kang et al. (2003) (Cell 113, p. 621, Fig 7) and Mueller et al. (2004) (J. Biol. Chem. 279, p. 11926, Fig 6), and (iii) CUE domains described by Prag et al. (2003) (Cell 113, p. 609, Fig 4). Accordingly, we believe that including additional sequence alignments in this study was unnecessary.

The sequence alignments presented in this study were conducted using only sequences with PDB accession numbers (i.e., those with known structures).

*2.*

*The structural data demonstrate that UBAh binds ubiquitin in vitro, but the functional role remains hypothetical. The manuscript should more explicitly acknowledge that the proposed role of UBAh in stalled ribosome recognition is based on structural compatibility rather than direct in vivo functional validation.*

As suggested, we have revised the sentence in the Abstract as follows:

Abstract: These findings demonstrate that UBAh specifically interacts with ubiquitin in vitro, providing structural insights into its potential role in recruiting HBS1L–PELO and SKI7 to stalled ribosomes.

Furthermore, clarifying phrases such as “in vitro,” “due to structural differences,” and “as revealed by sequence analysis” were added at appropriate locations throughout the manuscript to prevent misinterpretation.

*3.*

*The reported Kd of ∼50 μM is relatively weak compared to many high-affinity ubiquitin receptors. Given the high competition for ubiquitin in the cellular environment, the authors should discuss whether this affinity is sufficient for physiological function. Could avidity effects* *(via multi-valent interactions) or local concentration increases at the stalled ribosome compensate for this modest affinity?*

We appreciate the reviewer’s insightful comment regarding the moderate affinity (K_d_ ≈ 50 µM) observed for monoubiquitin. As noted in the Introduction, UBAh has been hypothesized to bind to the 40S subunit of ubiquitin, likely in the form of a polyubiquitin. Importantly, several CUE and UBA domains that bind monoubiquitin have been reported to recognize polyubiquitin with substantially higher affinity. For example, the UBA domain of E2-25K binds monoubiquitin with a K_d_ of ~400 µM, whereas its affinity for polyubiquitin is markedly stronger (K_d_ ≈ 27 µM). Thus, avidity effects arising from polyubiquitin on the 40S subunit surface are expected to enhance effective binding strength in vivo. Taken together, although the intrinsic affinity for monoubiquitin is moderate, the observed K_d_ (~50 µM) does not appear unphysiologically weak and is consistent with a functional role under cellular conditions.

We have added the following sentences to the Discussion section.

Page 20, line 11: Additionally, the Kd of UBAh for monoubiquitin is approximately 50 µM, which may appear moderate or relatively weak. However, because UBAh is proposed to bind polyubiquitin on the 40S subunit of stalled ribosomes in vivo, the avidity effects arising from polyubiquitin on the ribosomal surface are expected to enhance the effective binding strength by increasing the local ubiquitin concentration and promoting rapid rebinding to adjacent moieties. Indeed, several CUE and UBA domains that bind monoubiquitin have been reported to recognize polyubiquitin with substantially higher affinity [57, 67]. For example, the UBA domain of E2-25K, an ubiquitin-conjugating enzyme, binds monoubiquitin with a Kd of ~400 µM, whereas its affinity for polyubiquitin is markedly higher (Kd ~27 µM) [66]. Thus, the moderate affinity for monoubiquitin does not preclude a functional role under physiological conditions.

Minors

*1. The evolutionary comparison across species (e.g., yeast, nematodes, and metazoans) is interesting but largely speculative. It would be helpful to more explicitly label these interpretations as hypotheses rather than established conclusions.*

In *C. elegans*, the genome of which has been extensively characterized, no sequence corresponding to UBAh or any known structural domain has been identified. Therefore, its absence appears to be well supported rather than hypothetical. Because Hbs1 orthologs outside Nematoda retain the typical UBAh sequence, it is more plausible that the UBAh domain was lost in the *C. elegans* lineage during evolution rather acquired as a novel ubiquitin-binding domain.

Regarding fungi, we have revised the sentences as follows:

Page 22, line 8: Taken together, these observations suggest that fungal UBAh domains may exhibit some degree of structural divergence. Accordingly, the ubiquitin-binding capacity of individual UBAh domains warrants evaluation across fungal species.

*2. The discussion comparing direct ribosome binding in yeast versus ubiquitin-mediated recruitment in metazoans could be expanded slightly to address whether these modes might be mutually exclusive or potentially sequential.*

As suggested, we have added the following sentences:

Page 22, line 27: In contrast, because the UBAh domain of *S. cerevisiae* can bind directly to the small ribosomal subunit, there may have been an evolutionary stage in which ubiquitin-dependent and -independent modes of UBAh binding coexisted, functioning either mutually exclusively or sequentially. Such dual functionality may still be retained in some extant species. Further studies across diverse species are required to confirm this hypothesis.

*3. The section discussing acidic stretches and possible phosphorylation sites is intriguing but speculative. Minor rephrasing to emphasize these as future directions would improve balance.*

As suggested, we have added the following sentences:

Page 21, line 16: Further in vivo studies will be required to clarify the putative roles of the acidic stretches and Ser/Thr/Tyr-rich region. Their contributions are expected to vary depending on cellular conditions and operate either synergistically or in a complementary manner.

*4. Figure 1 is information-dense. Improving visual hierarchy or providing clearer panel descriptions would help the reader.*

As suggested, we have revised the figure to improve its clarity.

*5. Briefly clarify if global fitting was used for titration data or how residue-specific deviations were handled.*

Thank you for your helpful comments. We did not originally perform global fitting because of inadvertent oversight. We have now reanalyzed the titration data using global fitting, and the revised K_d_ value has been incorporated into the manuscript (50 µM → 52 µM). The graph in S11 Fig has also been updated accordingly.

We have revised the Materials and Methods and Results sections as follows:

Page 11, line 3: Global nonlinear regression analysis, assuming a single binding-site model, was performed using GraphPad Prism 6 based on titration data from three residues.

Page 19, line 22: Global fitting using data from Met84 (α1), Glu86 (α1), and Gly89 (α1/α2 loop) yielded a Kd of 52 µM (S11 Fig).

*6. (Page 14, Para 4): The phrase "These two ordered loops" is somewhat contradictory, as "ordered" usually implies rigidity, while loops are inherently flexible.*

*Probably "two well-defined loops" or "two structurally constrained loops" is better.*

We agree with this comment and have revised the phrase to “two well-defined loops” (page 14, line 15).
